# Recent progress in the improvement of hydrothermal stability of zeolites

**DOI:** 10.1039/d1sc01179k

**Published:** 2021-05-17

**Authors:** Raquel Simancas, Anand Chokkalingam, Shanmugam P. Elangovan, Zhendong Liu, Tsuneji Sano, Kenta Iyoki, Toru Wakihara, Tatsuya Okubo

**Affiliations:** Department of Chemical System Engineering, The University of Tokyo 7-3-1 Hongo, Bunkyo-ku Tokyo 13-8656 Japan wakihara@chemsys.t.u-tokyo.ac.jp okubo@chemsys.t.u-tokyo.ac.jp; Institute of Engineering Innovation, The University of Tokyo 2-11-16 Yayoi, Bunkyo-ku Tokyo 113-8656 Japan

## Abstract

Zeolites have been successfully employed in many catalytic reactions of industrial relevance. The severe conditions required in some processes, where high temperatures are frequently combined with the presence of steam, highlight the need of considering the evolution of the catalyst structure during the reaction. This review attempts to summarize the recently developed strategies to improve the hydrothermal framework stability of zeolites.

## Abstract 2

This review attempts to summarize the recently developed strategies to improve the hydrothermal framework stability of zeolites.

## Graphical abstract

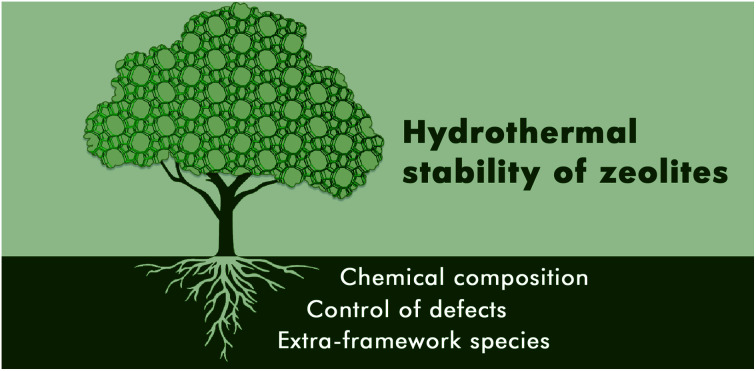

## 1. Introduction

More than 250 different zeolite framework types have been recognized by the International Zeolite Association (IZA).^1^ Microporous aluminosilicates are the major class of zeolites and their Si/Al ratio is a significant factor that influences properties such as ion-exchange capacity, thermal and hydrothermal stability, hydrophobicity, acidity, and catalytic activity. Most of the industrial applications are limited to less than ten structure types such as FAU, MFI, MOR, FER, LTA, CHA or *BEA.^2^

Zeolites are employed as catalysts in many different processes of industrial relevance, such as fluid catalytic cracking (FCC), selective catalytic reduction (SCR), methanol to hydrocarbons (MTH) reaction, dehydration of alcohols, alkylation, hydroxylation, and epoxidation, among others.^3^ Hydrothermal stability is an important characteristic of zeolites, which makes them one of the most widely employed materials for automobile applications *viz.*, exhaust purification, hydrocarbon trapping, diesel oxidation catalysis, SCR, *etc.*, where they are repeatedly subjected to processing under a high temperature in the presence of either trapped organics or water.^4,5^

For instance, a diesel particulate filter (DPF) needs to be treated above 650 °C on a regular basis to burn off all the particulate matter (PM) that got trapped within its porous network. Due to its close proximity to a DPF, the SCR catalyst is exposed periodically to heating treatments at varying temperatures in the presence of steam. In addition to the water obtained as a byproduct from the reduction reaction, diesel engine emissions may contain up to 10 vol% of H_2_O,^6^ whereas crude methanol used in the MTH reaction generally carried out at 400–450 °C contains around 17 wt% of H_2_O and this concentration increases during the reaction, where one water molecule is generated by each molecule of methanol converted.^7^

Moreover, before reactions, a pre-treatment of the catalyst at high temperatures is required for the regeneration of their catalytic properties. For instance, in the FCC process, the fast catalyst deactivation requires a continuous regeneration step carried out in the presence of steam at a temperature that can reach up to 760 °C.^8,9^ These processes make the catalyst vulnerable to structural degradation and hence possessing high hydrothermal stability is a must for its longer catalytic lifetime.

Hydrothermal treatments lead to chemical as well as structural changes which sometimes are irreversible depending on the stability of the zeolitic framework.^10^ Exposing the zeolite to those conditions causes the breaking of Si–O(H)–Al bonds by hydrolysis and extracting Al atoms (dealumination) from the structure, resulting in the formation of extra-framework aluminum species (EFAL) and decrease of the acidity of the catalysts.

Under severe dealumination conditions, excessive extraction of Al causes the removal of some Si atoms together with the Al atoms from the framework forming mesopores and structural defects that decrease the framework stability, resulting in a total or partial amorphization of the structure.^11–14^

Based on the knowledge gained from experimental results, several approaches have been developed to overcome the lack of hydrothermal stability of zeolitic materials.

### 1.1. Synthesis of high-silica zeolites

Zeolite synthesis requires the use of cations that compensate for the negative framework charge induced by the introduction of trivalent elements and at the same time fit the inner zeolite channels and cages. The use of bulky Organic Structure-Directing Agents (OSDAs) that can occupy large volume thus introducing relatively low charge density has allowed preparing zeolites with high adsorption capacities and high Si/Al ratios.^15–17^

### 1.2. Synthesis of a less defective framework

It is not surprising to find that high hydrophobic materials are more stable under steaming conditions. Fluoride-media synthesis has been proven as a method to reduce the formation of structural defects in the synthesis of pure and high-silica zeolites improving the structural stability.^18,19^

### 1.3. Mild dealumination treatments

Controlled mild hydrothermal treatment of zeolite Y, with a high Al content, results in a partial extraction of framework Al, forming EFAL species that remain in solids filling the pores, reducing the number of acid sites, and improving the hydrothermal stability and catalytic activity of the zeolite, known as ultrastable Y (USY).^20–22^ These EFAL species acting as Lewis acid sites, which have been located in close proximity to the Brønsted acid sites, enhance the stability of the framework.^23,24^

### 1.4. Healing of framework defects

The post-treatment of zeolites with silylating agents allows reducing the number of structural defects and internal and external silanol groups, increasing the hydrophobicity of the catalysts.^25–27^

### 1.5. Incorporation of extra-framework cations

The introduction of extra-framework cations reduces the acidity of the catalysts and at the same time stabilizes the Al in framework positions. The presence of extra-framework rare earth cations has shown a stabilizing effect under harsh conditions by preventing the dealumination of zeolite Y,^20,28,29^ whereas the interaction between extra-framework phosphate species and framework Al, mainly studied in the H-ZSM-5 zeolite, inhibits the dealumination under steaming conditions.^30–32^

The aim of this review is to consolidate newly developed strategies to improve zeolite stability.

After a brief introduction of the proposed dealumination mechanisms and theoretical studies about the process (Section 2), we will focus on the recent studies on the enhancement of zeolite framework stability. Section 3 covers the effect of the catalyst chemical composition and aluminum distribution on the framework stability and how to control them. Section 4 is focused on the presence of structural defects and the methods to lower their presence in the framework by direct and post-synthesis treatments. Section 5 summarizes the effect and strategies of the incorporation of extra-framework species, *viz.*, phosphorus, rare earth, alkaline and Cu^2+^ cations. In this review, we will focus on the improvement of aluminosilicate and silicate framework stability from the viewpoint of structural properties. The discussion about the stability of the catalysts in reactions is beyond the scope of this review, although some examples were included in the essay.

## 2. Zeolite dealumination mechanism

Several factors promote the dealumination of zeolites reducing the hydrothermal stability of the framework. Water accessibility has been proven as one of the main factors determining the reactivity of the Al sites.^33^ Water adsorption is promoted by the presence of hydrophilic centers, such as silanol groups or framework defects and cations balancing the negative framework charge induced by the introduction of trivalent elements.^34^

Sano *et al.*^35,36^ showed linear correlation between adsorbed water molecules and the content of framework Al in the H-ZSM-5 zeolite, and a dealumination rate dependence on the content of Al under hydrothermal treatments at 600–800 °C.

Agostini *et al.*^37^ employed *in situ* time-resolved XRPD and TG, *in situ* Al K-edge XAS, and *ex situ*^27^Al magic angle spinning (MAS) NMR to determine the dealumination mechanism of zeolite Y during steaming at 600 °C. Their results indicate that the formation of extra-framework Al species mainly occurs during the cooling stage (177–227 °C), when water molecules are capable of diffusing through the zeolite framework.

Muller *et al.*^38^ studied the dealumination of zeolites *BEA, MOR, MFI and FER by thermal treatment, leaching with oxalic acid and treatment with SiCl_4_ using ^1^H, ^29^Si and ^27^Al MAS NMR. The zeolite structure, Si/Al ratio and crystal size were found as the main factors determining the dealumination behavior. Furthermore, the presence of defect sites formed during crystallization or calcination results in a more flexible framework that shows less stability against dealumination.

Almutairi *et al.*^39^ showed the difference in the stability of the zeolite H-ZSM-5 when it was treated at 400–600 °C for 6 h under mild (8.2 × 10^−5^ mol min^−1^) and severe (3.3 × 10^−4^ mol min^−1^) steaming conditions. While the removal of framework Al was not observed under mild conditions, an increase in the steam flow resulted in severe dealumination, decrease of Brønsted acid sites, and formation of mesoporosity.

In another study, Iyoki *et al.*^12^ found the formation of an amorphous layer on the surface of zeolite Y during hydrothermal treatments which covers the zeolite particles preventing the adsorption of Ar and even the particle core does not suffer from a severe degradation and dealumination.

A controlled dealumination has been used to enhance zeolite stability and obtain high-silica materials from zeolites that can only be obtained with a high Al content.^40–42^ But an excessive extraction of Al can lead to an undesired effect during the reaction and regeneration of the catalysts, underlining the importance of understanding the dealumination mechanism and avoiding it when it is undesired. The dealumination process has been studied by different characterization techniques, such as solid-state ^27^Al MAS NMR and Fourier transform infrared spectroscopy (FT-IR), which are powerful and widely used techniques employed in the study of Al atoms.^30,39,43^

Ristanovic *et al.*^44^ studied the differences between mild (500 °C) and severe (700 °C) steaming treatments on ZSM-5 zeolites by measuring the local turnover rates of the oligomerization of furfuryl alcohol using the concept of nanometer accuracy by stochastic chemical reactions (NASCA) microscopy. Mild steamed zeolites showed a highly heterogeneous distribution of acid sites caused by the formation of extra-framework aluminum species and mesoporous defects in the surface region, while the severe treatment caused a significant loss of Brønsted acid sites.

Perea *et al.*^45^ employed atom probe tomography to determine the Al–Al bond length in the ZSM-5 zeolite, observing a non-random distribution of the Al atoms before and after steaming treatment, concluding that the porous network acts as a highway for Al transport.

**Fig. 1:**
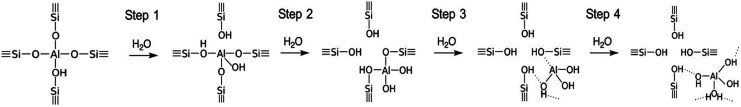
Suggested schema of Al extraction from the zeolitic structure.^47,48^ Reproduced from ref. 48 with permission from the Royal Society of Chemistry.

Although the dealumination mechanism has not been fully understood, the experimental results indicate that a severe treatment at high temperatures in the presence of steam leads to the extraction of the Al atoms and the formation of silanol defects.^46^Fig. 1 shows the proposed four step dealumination mechanism.^46–48^

Computational simulation has been proven as a very important tool that combined with the experimental results allowed rationalizing the reaction mechanism. Recently, several theoretical studies have been carried out to elucidate the initial steps of the dealumination mechanism.

Silaghi *et al.*^49^ reported a dealumination mechanism where one water molecule is adsorbed on the Al atom which adopts a pentahedral coordination leading to bond breaking and resulting in the formation of tetrahedral Al partially bonded to the structure. This initiation mechanism agrees with experimental results; besides, it was confirmed in four different zeolitic frameworks, CHA, MOR, FAU and MFI.

**Fig. 2:**
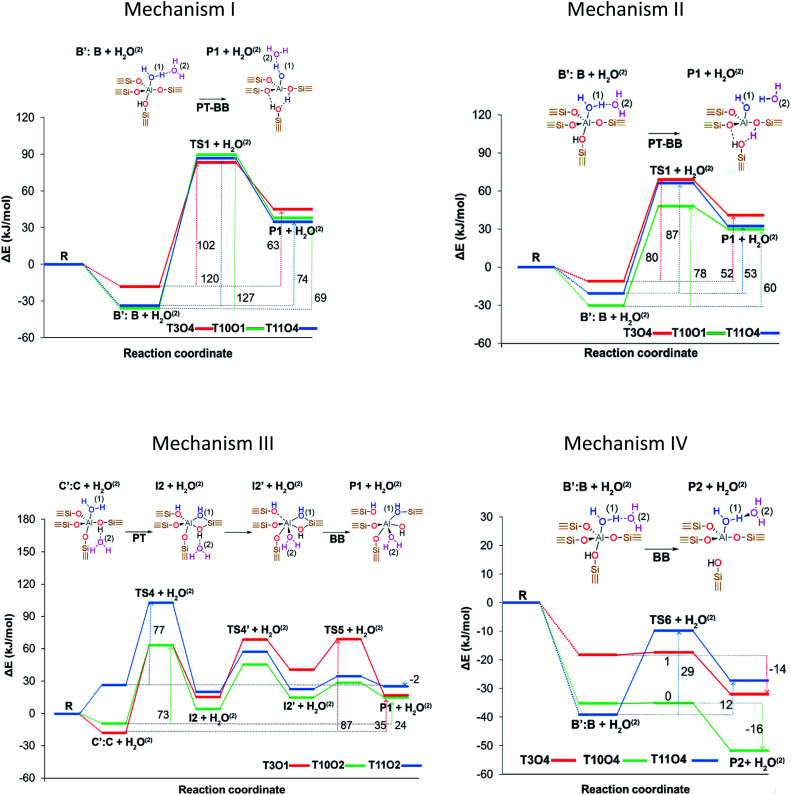
Proposed initiation dealumination mechanisms involving two water molecules. Reprinted with permission from ref. 33. Copyright (2019) American Chemical Society.

Stanciakova *et al.*^33^ proposed four mechanisms involving two water molecules in zeolite MFI as shown in Fig. 2, where the water–water interaction affects the reaction thermodynamics. Prior to any step, the water molecules should be rearranged from stable adsorption modes to active fewer stable modes; therefore, the facility of this rearrangement could have an important influence on the dealumination process. Mechanism I is analogous to that reported by Silaghi *et al.*^49^ for one water molecule, while mechanism II is a modified version following a more energetically favorable pathway. In mechanism III the second water molecule interacts with the zeolitic framework before the Al–O(H)–Si bond breaking, whereas mechanism IV is thermodynamically preferred resulting in P2 species that could be reactants in mechanisms I and II. The authors concluded that the water accessibility to the Al sites and treatment temperature are the main factors determining the reactivity of the acid sites.

**Fig. 3:**
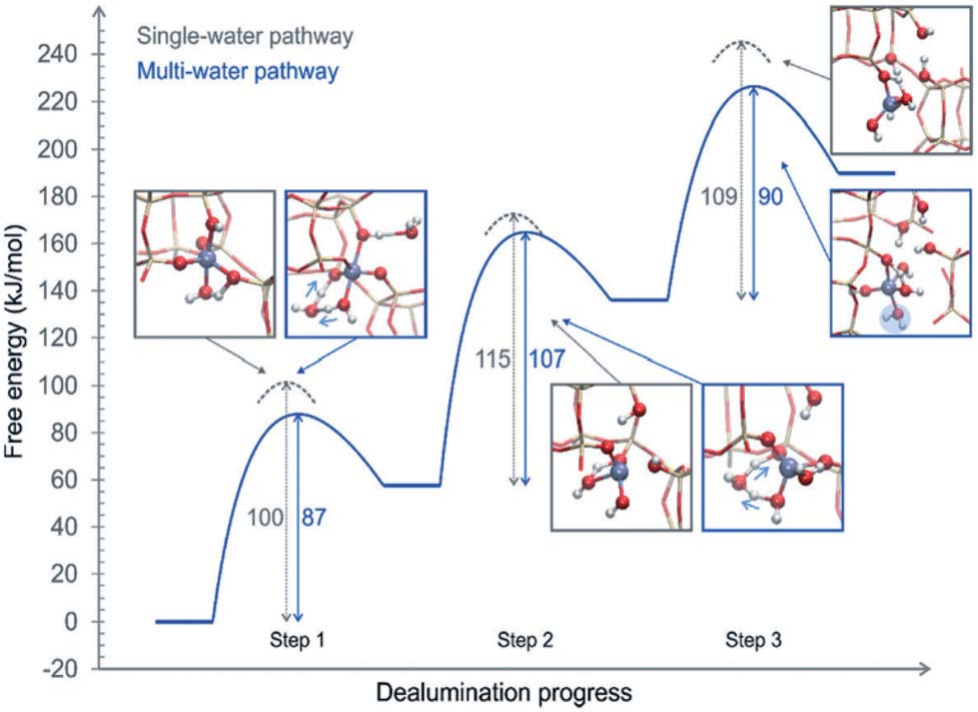
Free energy diagram of a complete dealumination reaction in a CHA-type framework as obtained from DFT-MD umbrella simulations. Reproduced from ref. 48 with permission from the Royal Society of Chemistry.

The above proposed mechanisms were only focused on the first hydrolysis step studying the system from a static approach. Nielsen *et al.*^48^ studied the dealumination process in a CHA-type framework by using DFT-based molecular dynamics (DFT-MD) simulations to solve the problem under realistic conditions (atmospheric steam pressure at 450 °C). The free energy barriers of Al–O hydrolysis were calculated for the mechanism where a single molecule was present in every step of the dealumination process, as shown in Fig. 1, and for the system containing four water molecules in the unit cell, as shown in Fig. 3. The authors concluded that water molecules cooperate in the dealumination process by stabilizing the transition state of each step.

## 3. Improvement of thermal/hydrothermal stability by controlling chemical composition and aluminum distribution

High silica zeolites (*i.e.* Si/Al > 100) are favored in many applications as they show high hydrothermal stability with the exception of ion-exchange processes where a low Si/Al ratio is preferred. Aluminum gets removed from its framework position, which is termed dealumination and occurs due to high temperature treatments either with or without steam and is found to be the major cause of the structural instability of various zeolites.^13,14^

### 3.1. Control of chemical composition by synthesis and post-modification strategies

In order to retain structural integrity and improve the hydrothermal stability of zeolites, researchers focused mainly on the following techniques, *i.e.* direct synthesis of high-silica zeolites using OSDAs, synthesis using fluoride media, dry gel conversion technique^15,18,50–52^ and post-synthetic modifications using mild dealumination techniques such as SiCl_4_ treatment, mild acid treatment, steaming, or combination thereof.^13,14,53–55^

#### 3.1.1. Direct synthesis

Aluminosilicate zeolites are synthesized using different sources of silica, aluminum, charge compensating alkali cations, mostly Na^+^ or K^+^, and mineralizer, OH^−^ or F^−^, in the presence or absence of OSDAs. The formed zeolites will bear a negative charge depending on the content of Al present in the zeolite framework, which is usually charge compensated by alkali cations that can be later exchanged with any other cations, mostly mono- or divalent elements, based on the requirements.

A large number of AlO_4/2_^−^ ions are required to stabilize the aluminosilicate precursor and lead to the formation of aluminum rich zeolites. Due to the larger content of Al, more negative charges on the framework can be created, which in turn act as an ion-exchange site, an active site for many applications. However, the presence of more aluminum leads to easy dealumination and causes structural collapse, and hence, high-silica zeolites are preferred for applications involving high temperatures in the presence of water vapor.

Researchers found that quaternary ammonium cations due to their bulky nature occupy more space and there is no need for AlO_4/2_^−^ ions for charge compensation and results in obtaining high-silica zeolites. A major breakthrough has recently been made to synthesize high silica zeolites, *viz.*, *BEA, MTW, MSE, TON, and so on, without the use of OSDAs but with the addition of zeolite seeds.^13,40,56–59^

Although there are many articles and reviews dealing with the synthesis of high-silica zeolites,^15,50,51,60–64^ recent study on their hydrothermal stability is scarce; however, to get an overall view of high-silica zeolites including trends in the zeolite synthesis, researchers are encouraged to read those reviews and articles.

In the zeolite synthesis under fluoride media, where the supersaturation of crystallizing species is relatively lower, the formation of metastable phases is largely reduced. It offers the advantages of narrow crystal size distribution, fewer defects and ordered crystallization. Fluoride sources such as HF, NH_4_F, NaF and KF were used, and F^−^ ions get incorporated into the zeolite framework by replacing OH^−^ ions. This enhances the hydrophobicity of the material, which certainly contributed to the increase in the hydrothermal stability of the obtained zeolites.^65–68^

The synthesis of hydrothermally stable, all silica zeolites such as BEA* and CHA became possible by the introduction of fluoride ions.^18^ Kato *et al.* studied the synthesis of mordenite in fluoride media and explained its stability based on the ordered distribution of Al ascribed to the presence of F^−^ ions in the reactant.^69^ The effect of zeolite synthesis under fluoride media in limiting defect formation is discussed in detail in Section 4.2.

#### 3.1.2. Post-synthetic modifications

Improving the hydrothermal stability of zeolites is also possible by post-synthetic modifications such as acid treatment, to increase the Si/Al ratio, followed by steaming to repair structural defects *via* silicon migration.^43,54,55^ In addition to acid treatment, there are some reports on the dealumination of zeolites by numerous techniques *viz.*, SiCl_4_ treatment, ammonium hexafluorosilicate, *etc.*^14,70^ Recently, the improvement of the hydrothermal stability of an Al-rich large pore zeolite (OSDA-free synthesized) was achieved by this approach.^13^ Chung *et al.* studied the hydrothermal stability of both synthetic and natural mordenite under simulated lean NO_*x*_ wet conditions and found that the hydrothermal stability becomes stronger as the Si/Al ratio increases.^71^

Lercher and co-workers found that the extra-framework Al species in ion-exchange positions, that were formed as a result of the steaming treatment, played a crucial role in enhancing the hydrothermal stability of H-*BEA zeolites *via* controlled dealumination and lattice stabilization.^55^

Post-synthetic dealumination using a mild acid on the as-synthesized zeolite *BEA (with an occluded OSDA) was reported to enhance its hydrothermal stability, resulting in a Si/Al > 1000 and the formed silanol defects were found to be cured by calcination.^72^ Weckhuysen and co-workers suggested a post-synthetic pressure-controlled dealumination treatment to produce hydrothermally stable, reactive zeolite catalysts.^33^ A versatile dealumination strategy was also proposed by Shi *et al.* to stabilize Al-rich zeolites through interaction between the zeolite framework Al and aluminum sulfate.^73^ Although high silica zeolites are hydrothermally more stable than aluminum rich zeolites, which are more susceptible to dealumination, the lack of exchangeable cation sites makes the former unsuitable for numerous applications. Under such circumstances, it becomes necessary to select zeolites with an optimum Si/Al ratio to maintain a balance between activity and hydrothermal stability.^74–76^

In this context, attempts were even made to incorporate Al into high silica zeolites under fluoride media using zeolite crystals as an aluminum source.^19^ The effects of hydrothermal treatment on metal exchanged zeolites were studied by several authors.^77–79^ With an increase in hydrothermal aging temperature, the structure of zeolites with a higher Cu/Al ratio could easily deteriorate.^76,80^ As reported, there exists a threshold for the Cu/Al ratio (*i.e.* 0.35), above which the effect of aging will lead to severe structural disintegration.

Such an effect is not only limited to the Cu/Al ratio as the CuO cluster formation beyond a certain threshold may even lead to hydrothermal aging^81,82^ and the removal of CuO clusters by washing with a dilute acid followed by ammonium nitrate aqueous solution seemed to improve the hydrothermal stability of these zeolites.^83^ Recently, the coating of pure silica on the surface of the zeolite was found to not only increase the overall Si/Al ratio but also act as a shield to protect the inner aluminosilicates from dealumination thus endowing it with high hydrothermal stability.^84^

### 3.2. Effect of Al distribution

The contents and locations of Al in the zeolite frameworks are the key factors determining the physicochemical properties of zeolites.^85–90^ The location of Al in the framework plays a vital role in determining the stabilization of exchanged cations, especially di or trivalent ions, and in turn the activity or stability of the catalyst.^91–94^ Al distribution mainly depends on the Si/Al ratio,^95,96^ and it controls not only activity but also hydrothermal stability. Yokoi *et al.*^88^ reported that the Al atoms located at the intersection of the ZSM-5 zeolite exhibited a shorter catalytic lifetime by investigating the correlation between the location of Al in the framework and catalytic activity/stability. They used high resolution ^27^Al MAS NMR and ^27^Al MQMAS NMR techniques to examine the location of Al on the ZSM-5 zeolite synthesized using various OSDAs *viz.*, tetrapropylammonium cations, dipropylamine, hexamethylenimine or cyclohexylamine with or without Na^+^ cations.

**Fig. 4:**
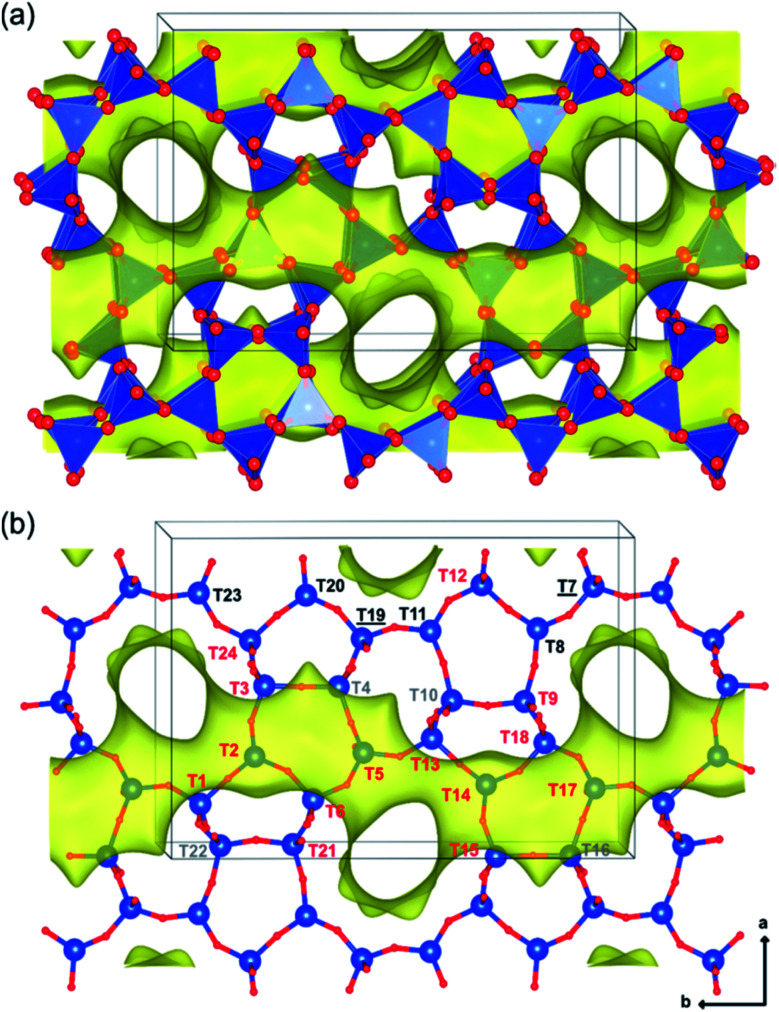
Illustrations of the monoclinic MFI structure, viewed perpendicularly to the straight channels, as tetrahedral units (a) and as bonded atoms (b). The 24 different tetrahedral framework sites are indicated in part b and are denoted in red (intersections), gray (sinusoidal channel only), black (straight channel only), and black underlined (shared by both straight and sinusoidal channels but not located at the intersection). Reprinted with permission from ref. 43. Copyright (2018) American Chemical Society.

**Fig. 5:**
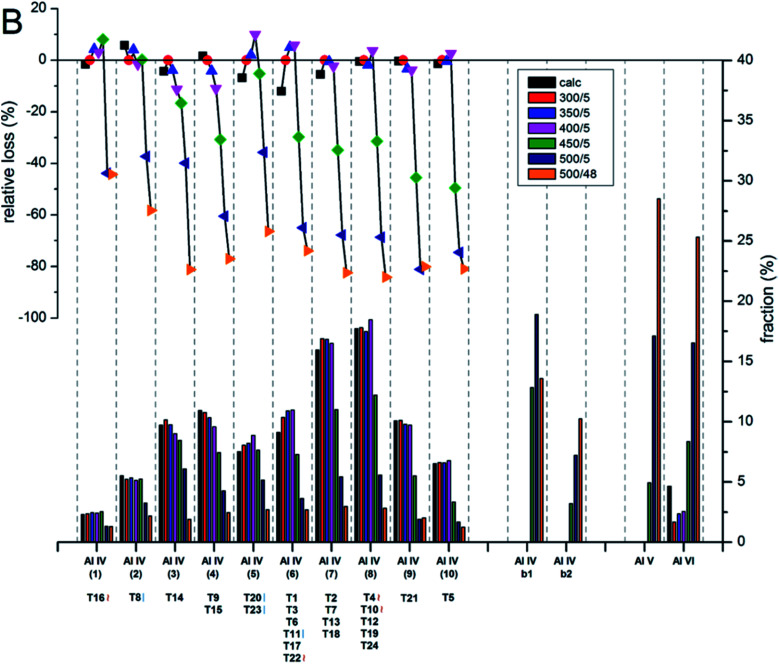
Evolution of the intensities for the different Al sites after the steam treatment of sample B (Si/Al = 40) determined and quantified by ^27^Al MAS NMR at 22.3 T. The lower bars show the fraction of Al intensity for the individual sites, whereas the symbols in the upper part indicate the relative loss of Al intensity upon steam treatment at different temperatures. Reprinted with permission from ref. 43. Copyright (2018) American Chemical Society.

Holzinger *et al.*^43^ reported a similar observation by investigating the aluminum distribution of four different commercial H-ZSM-5 samples (Samples A, B, C and D of Si/Al ratios of 15, 40, 50 and 140, respectively) before and after steam treatment using the ultrahigh field ^27^Al MAS NMR technique. In agreement with theoretical studies,^90,97^ they have arrived at the conclusion, using the normalized data obtained from ^27^Al NMR for sample B after steaming treatment at 500 °C for about 48 h, that Al in the T sites of the straight and the sinusoidal channels is most stable (T16, T8, T20, and T23; lost only 44–66% of their initial intensity), while framework sites facing the intersections are most prone to dealumination, where more than 77% of Al has been removed (*cf.*Fig. 4 and 5).

In addition, Yokoi *et al.* found a correlation between the relative crystallinity after steam treatment carried out at 800 °C in 40 vol% steam and the Q4 (*n*Al) and Q3 (*n*Al) proportions in the parent CHA samples and concluded that Q4 (2Al) is more prone to dealumination compared to Q4 (1Al).^98^ Davis and co-workers also reported that compared to isolated Al species, the presence of higher proportions of pairing Al species in CHA-type zeolites led to the production of heavier coke in the MTO reaction.^99^

The location and distribution of framework Al atoms dramatically affect the catalytic activity and selectivity in protonic as well as transition metal ion-containing zeolite catalysts.^100–103^ The regulation of the Al location or the acid distribution in the framework has proven beneficial for improving the hydrothermal stability of zeolites, thereby making them an industrially useful catalyst.

Zeolite researchers have tackled controlling Al distribution especially on MFI zeolites and partially on *BEA, CHA and AEI; however, the effect of the distribution of Al atoms in the zeolite framework on the hydrothermal stability of most of the zeolites has not been completely understood and it is still a challenging issue to be resolved.

## 4. Improvement of thermal/hydrothermal stability by decreasing structural defects

### 4.1. Structure of defects

The term “defect” in a zeolite framework predominantly refers to silanol (SiOH) groups and depending on their location they may be classified as framework or surface defects. Relative to the chemical proportions of the initial multicomponent reactant mixture and its treatment conditions they appear in all shapes and sizes which can be quantified by a combination of characterization techniques such as physisorption, X-ray diffraction (XRD), NMR spectroscopy, vibrational spectroscopy, electronic structure mapping and numerical solution calculations.

The influence of synthesis parameters and post-synthesis treatments on the formation and annealing of various kinds of defects in high-silica MFI zeolites has been established systematically using high-resolution solid-state ^29^Si NMR.^104^ While the cause of their formation during synthesis and healing after post-synthesis treatments is yet to be fully understood, different models were proposed for their types using various zeolite frameworks (MFI, MTW, AFI and -SVR) by Brunklaus *et al.*^105^ with multiple quantum NMR spectroscopy.

The authors observed connectivity defects among MFI, MTW and AFI while -SVR contained ordered vacancy defects. It was suggested that the presence of merged six rings preferentially formed connectivity defects in three zeolites while the fourth lacking it contained vacancy defects. Furthermore, they implied that connectivity defects are preferred over vacancies because the former healed by condensation upon calcination while the latter was not.

**Fig. 6:**
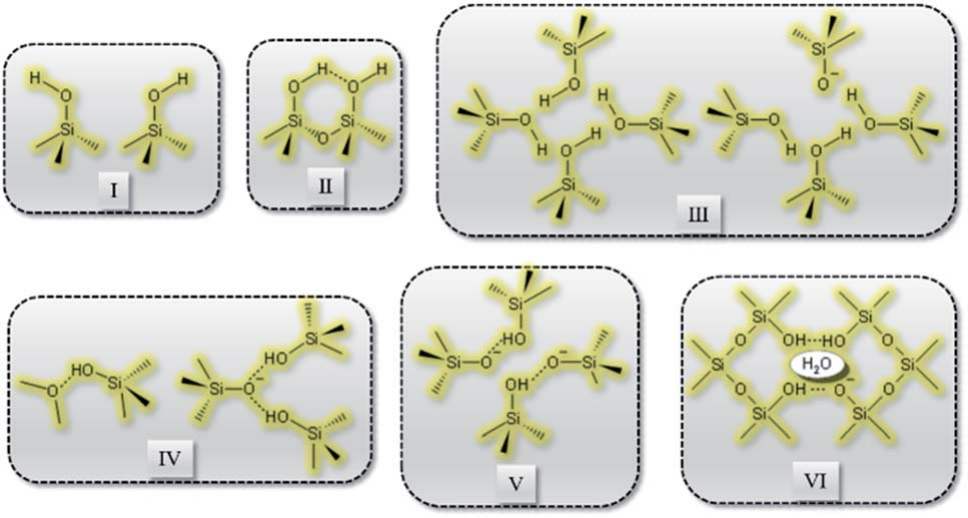
Types of defects: (I) isolated, (II) paired silanol, (III) silanol nests, (IV) combined silanol groups, (V) associated pairs and (VI) clustered pairs with stabilizing H_2_O.

Recently, Grosskreuz *et al.*^106^ gave an overview of previously established defect models by investigating the alteration of different framework defects in MFI zeolites. The authors suggested that defects for MFI-type zeolites fall within one of the six types shown in Fig. 6. It was established that upon calcination at high temperatures most of the defects get cured, whereas an imperfect condensation leads to empty T-sites in the case of silanol nests. Avoiding defects altogether or healing them if present is crucial and failing to do so would compromise the framework stability and performance of zeolites. There are several studies in the open literature that report different techniques to address the issues related to defect formation in zeolites which will be discussed in the sections to follow.

### 4.2. Synthesis of zeolites in fluoride media

Conducting the synthesis in fluoride media and post-synthesis silylation are well-known techniques used to decrease the defects in the zeolite structure. The practice of using fluoride ions as a mineralizing agent dates back to 1978 when Flanigen and Patton successfully attempted zeolite preparation in near neutral synthesis media.^107^ Preparing zeolites in the fluoride medium was found effective in mitigating defects when Chézeau *et al.* reported the synthesis of a highly siliceous MFI-type zeolite using fluoride anions as the mineralizer.^108^ Though this technique is instrumental in producing pure or high-silica defect-free zeolites, employing them for preparing aluminosilicate zeolites remained challenging until Moteki and Lobo reported the synthesis of ITW-, CHA-, *BEA- and STT-type zeolites in fluoride media using a FAU-type zeolite as an effective aluminum (Al) source to control the defects as well as the Si/Al ratio.^19^

Recently, fluoride media were used to prepare ZSM-5 model crystals with fewer defects and a near perfect structure–diffusion–activity relationship was established using the same in MTO catalysis. Using this model, it was shown that if the defects were fewer a molecule within the microporous network attains diffusional supermobility or levitation and passes through at least 5 to 6 channel intersections before reaching the nearest acid site thus enhancing the catalytic activity and selectivity.^109^

Previously, the synthesis of zeolites and zeo-types employing fluoride media was achieved in the pH range 5–9. Preparing zeolites at pH below 5 required seed assistance until the pioneering work of Shi and coworkers opened new possibilities by producing zeolites above the isoelectric point of gel particles in the pH range 2.3–5.^110^ An *in situ* IR spectroscopy study on ZSM-5 nanocrystals conducted by Grahn and coworkers showed that lowering the density of internal defects using the fluoride route enhanced their lifetime to 6 times longer than that of the crystals prepared in traditional hydroxyl medium in the methanol to hydrocarbons (MTH) reaction. The abundance of internal defect sites present in the sample was found to accelerate coke deposition in its channel system causing hindered diffusion and percolation effects leading to faster deactivation than the fluoride based one.^111^

The reports on fluoride-media syntheses aiding the enhancement of catalytic activity, stability and selectivity of zeolites by lowering the defect sites are vast; however, studies exploring their influence on hydrothermal stability are lacking. Conclusions from a recent study investigating such an influence by comparing the hydrothermal stability of ZSM-5 crystals prepared from fluoride and hydroxide media reported by the same group might explain such lacking.

The steam treatment of the ZSM-5 crystals was performed at 1000 °C for 32 h and that prepared from fluoride media exhibited relatively higher framework stability (established from the relative crystallinity) than its hydroxide counterpart. However, it's intriguing to note that the dealumination occurred with comparable rapidity in both cases, necessitating the need for further investigations to understand the role of fluoride anions in stabilizing the framework structure.^112^

### 4.3. Treatment of zeolites with a silylating agent (hydrophobization)

Similar to fluoride media synthesis (discussed in Section 4.2), post-synthetic treatment with silylating agents is a well-established method to overcome the influence of defects on the framework stability of zeolites, and even a few studies have identified the latter to be better. One such study by Prodinger *et al.* states that the role of fluoride anions as a hydrothermal stability enhancer (*via* defect minimization) was effective only when the concentration of acid sites is lower *i.e.* on high silica zeolites (Si/Al > 100) and somehow failed to prevent hydrolytic degeneration in zeolites (Si/Al < 50) with a higher concentration of acid sites. Under such circumstances, a selective hydrophobization restricted to the external surface by post-synthetic silylation using long-chain alkyl chlorosilanes is shown to extend the lifetime of defect free Al rich zeolites.^113,114^

Following that study, Vu *et al.* reported the stability enhancing effect of mono-, di- and tri-chlorosilanes post-synthetically on zeolite Y by systematically varying their alkyl chain lengths for the first time in acidic aqueous media under aqueous-phase processing conditions (*i.e.* pH ≈ 2, 200 °C and autogenous pressure). Among the silylating agents studied, methyltrichlorosilane was found to improve the hydrothermal stability of zeolite Y significantly.

Unfortunately, a general flaw (*i.e.* the narrowing effect of the silylating agents on zeolite pores causing their micropore volume to shrink) encountered with all the post-synthetic silylations reported so far was observed in this study as well.^115^

Although most of the methods discussed so far in this review were able to suppress structural defects and enhance the hydrothermal stability of zeolites to withstand steaming treatment up to a maximum temperature of 850 °C, pushing the limit beyond 1100 °C remained challenging until the following two methods (*i.e.* synthesis in the presence of alcohol^116^ and self-defect healing^117^) were established.

Yoshioka *et al.* recently unfolded a rapid synthesis technique for preparing a ZSM-5 zeolite in just 2 h by using 1-butanol as an organic additive instead of the usually employed tetrapropylammonium (TPA) cation as the OSDA. Since alcohols are essentially electroneutral and withstand very high synthesis temperatures they are known to generate far fewer defects than TPA cations. Notably, ZSM-5 synthesized in alcoholic media has their Al distributed in a unique fashion leading to a superior catalytic lifetime as well. Compared to TPA, the ZSM-5 synthesized from 1-butanol retained not only 74% of its crystallinity after undergoing a hydrothermal stability test at 1100 °C but also possessed Al atoms in their framework indicating that the synthesis of zeolites in alcohol medium has resulted in widening of the synthesis temperature window and suppression of silanol defects and dealumination.^116^

### 4.4. Self-defect healing

**Fig. 7:**
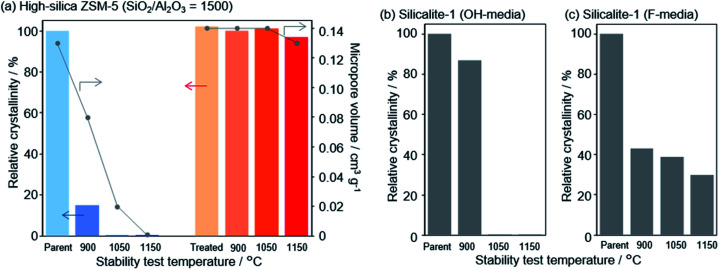
Effect of stabilization treatment on crystallinity and micropore volume. Changes in relative crystallinities and micropore volume of (a) parent and treated high-silica ZSM-5 (SiO_2_/Al_2_O_3_ = 1500) after the stability test and changes in the relative crystallinity of pure-silica ZSM-5 (silicalite-1) synthesized in (b) hydroxide and (c) fluoride media. Reprinted with permission from ref. 117. Copyright (2020) American Chemical Society.

In a recent finding, Wakihara and co-workers were able to stabilize the framework of industrially important zeolites (*BEA, MFI and MOR) with three different topologies to withstand extremely harsh steaming conditions exceeding 1100 °C.^117^ Using a simple and general cooperative liquid-mediated method that involves mutual assistance of pore filling hydroxide and fluoride species, the authors post-synthetically have achieved silicate migration, inducing defect-healing in these zeolites. Unlike many post-synthetic silylation processes,^113–115^ this new process does not require additional silylating agents, which tends to reduce micropore volume to a significant extent *via* pore blockage. Fig. 7 shows the highlight of this technique to heal defects to an extreme extent by comparing the relative crystallinities and micropore volumes of the high-silica ZSM-5 zeolite (SiO_2_/Al_2_O_3_ = 1500) both before and after the cooperative liquid-mediated treatment.

**Fig. 8:**
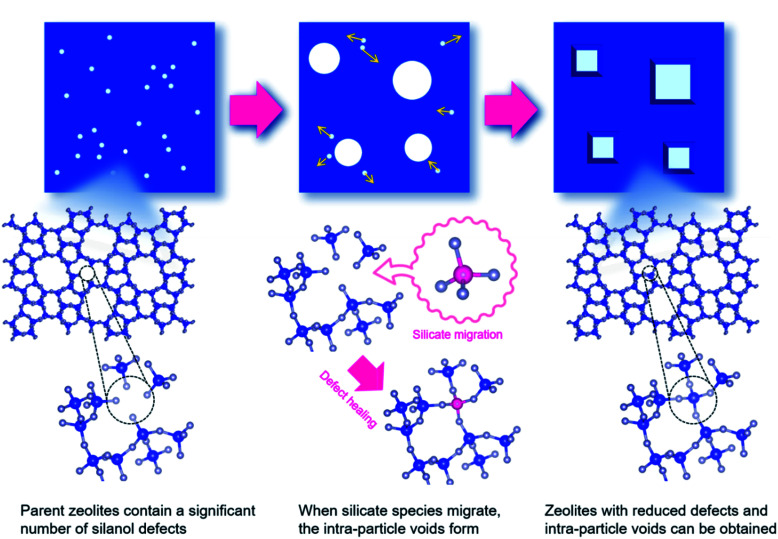
Proposed mechanism for self-defect healing. Reprinted with permission from ref. 117. Copyright (2020) American Chemical Society.

At 900 °C the structure of the untreated ZSM-5 degrades completely while that of the treated ZSM-5 remained intact even a treatment temperature of 1150 °C. Even the pure silica ZSM-5 (Silicalite-1) prepared from hydroxide and fluoride media lost its structural integrity at 900 °C. Also, a self-defect healing mechanism involving the migration of silicate species for this method is proposed in Fig. 8. This new post-synthetic method is found beneficial for improving the hydrothermal stability of different high-silica zeolites falling within a certain range of SiO_2_/Al_2_O_3_. However, the context of improving the hydrothermal stability of low-silica zeolites using this method might require furthermore modifications to address the dominance of dealumination under such high-temperature steaming conditions.^117^

## 5. Improvement of hydrothermal stability by the introduction of extra-framework species

### 5.1. Incorporation of extra-framework phosphorus species

The improvement of the hydrothermal stability of zeolites by the introduction of extra-framework phosphorus species is well-known, having been studied in many zeolites, especially in MFI and FAU, due to their important industrial applications in catalytic cracking or in the methanol to hydrocarbon reactions.^30,118,119^

The incorporation of extra-framework phosphorus species not only enhances the stability of the zeolitic framework under high temperatures in the presence of steam, but also reduces the number of acid sites, and their strength, because the interaction of phosphates with strong Brønsted acid sites is preferred over weak acid sites.^31,32,120^ This effect on the acidity has an important influence on the catalytic performance allowing the control of product selectivity.^121^

The presence of phosphorus also avoids the collapse of the structure, showing low variation of the micropore volume before and after steaming treatment of P-MFI.^30^

In Section 2, it was shown that after the treatment of the zeolites under high temperature in the presence of steam, a dealumination process occurs. Zeolites modified with P suffered from less dealumination, preserving more Al in framework positions. Nevertheless, it has been reported that there is an optimum P content at which the minimum number of acid sites is lost keeping the maximum stability for the framework aluminum and catalytic performance.^30,122^

#### 5.1.1. Methods to introduce extra-framework phosphorus species

The phosphorus species are commonly incorporated by wet impregnation using an aqueous solution of H_3_PO_4_, NH_4_H_2_PO_4_ or (NH_4_)_2_HPO_4_.^30,31,123^ Indeed, the phosphorus sources employed in the treatment could have an important influence on the phosphated zeolite properties. Recently, Bao *et al.*^124^ studied P-MCM-22 (MWW-type) zeolites prepared by impregnation using three different P sources, H_3_PO_4_, tributylphosphine and phenylphosphonic acid. All samples suffered from a mild dealumination mainly of the Al located in the T2 site, which is located in the supercages and surface pocket, causing a decrease of the catalyst acidity; however, the sample treated with tributylphosphine showed the highest relative crystallinity after impregnation and the best stability in the *n*-hexane cracking reaction. Although the impregnation method is the most used preparation, it could proceed with preferential incorporation of phosphorus on the external surface.

Gao *et al.*^125^ compared a P-ZSM-5 zeolite prepared by the impregnation method with a series of samples with different P contents obtained by using the hydrothermal dispersion method, where a mixture of H-ZSM-5 and (NH_4_)_2_HPO_4_ was heated at 140 °C and 0.3 MPa for 2 h. The hydrothermal dispersion enhances the P diffusion through the zeolitic channels, improving the catalytic performance in the butene cracking of the phosphated zeolites. Other reported methods are sample reflux with an aqueous solution of H_3_PO_4_ or in an *n*-octane solution of trimethylphosphite,^126^ and the gas-phase deposition using as precursor trimethylphosphite^127^ or trimethylphosphine,^128^ but they are less used.

Most of the incorporated phosphate species by using the above treatments are monomeric, di- or small chain polyphosphates that after the required calcination step evolve into large polyphosphates which have no interaction with the zeolite framework.

**Fig. 9:**
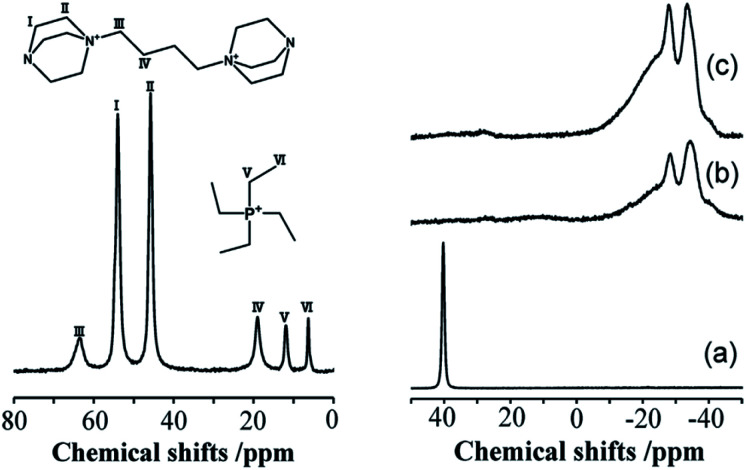
(Left) ^13^C CP/MAS NMR spectrum of the as-synthesized P-modified AFX and (right) ^31^P MAS NMR spectra of P-modified AFX zeolites: (a) as-synthesized (run 10), (b) calcined (run 6), and (c) calcined (run 10). Reprinted from ref. 137, with permission from Elsevier.

The incorporation of well-dispersed P-species during the zeolite synthesis has been recently explored by using a phosphorus-containing OSDA. This method allowed obtaining new zeolitic structures^129–132^ and introducing phosphorus into small pore materials that cannot be achieved by standard post-synthesis treatments because of the limited diffusion of phosphates through 8R windows. Small pore zeolites AEI,^133–135^ CHA,^136^ AFX,^137^ LEV,^122^ GME^138^ and AFX/CHA^139^ have been prepared by using a dual-template method where the typical OSDA employed in the crystallization of the zeolite was mixed with tetraethylphosphonium or tetramethylphosphonium hydroxide (Table 1). The presence of tetraalkylammonium (N-OSDA) and tetraalkylphosphonium (P-OSDA) can be confirmed by ^13^C CP/MAS NMR spectroscopy, and in the case of the AFX zeolite the characteristic shifts of both organic molecules can be distinguished in the ^13^C CP/MAS NMR spectrum (Fig. 9 left). After calcination, the N-OSDA is decomposed to NO_*x*_, CO_2_ and H_2_O molecules that can diffuse through the zeolite channels while the P-OSDA decomposes/oxidizes generating P_*x*_O_*y*_ species that remain in the pores and cavities of the material.

**Table 1:** OSDAs used in the preparation of small pore zeolites by the dual-template method

Zeolite	N-OSDA	P-OSDA
CHA^136^	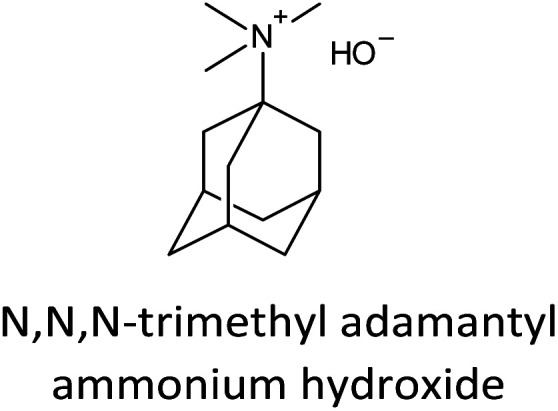	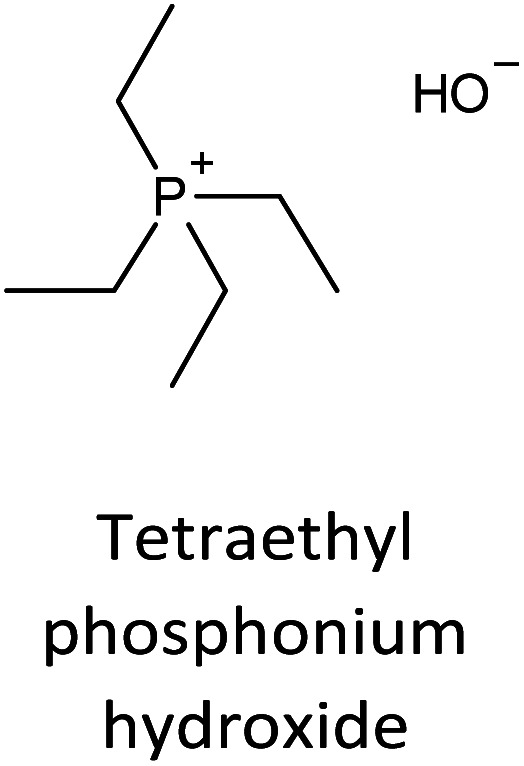
AEI^134^	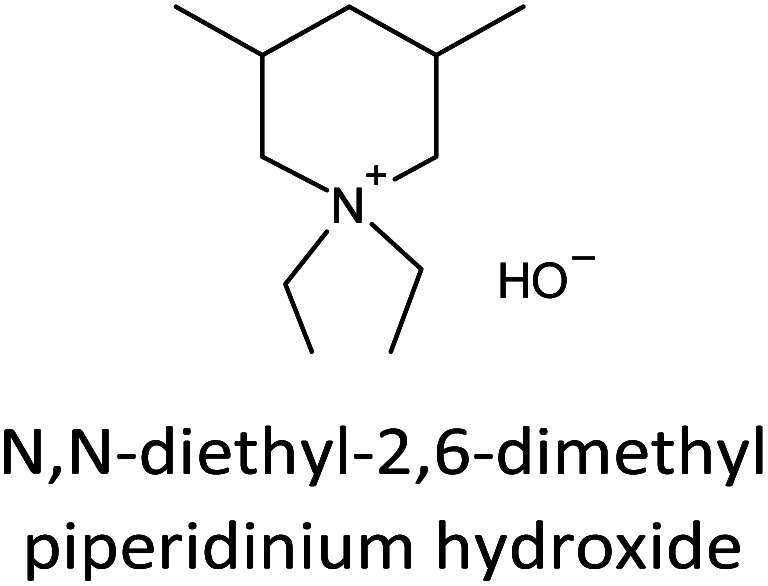	
GME^138^	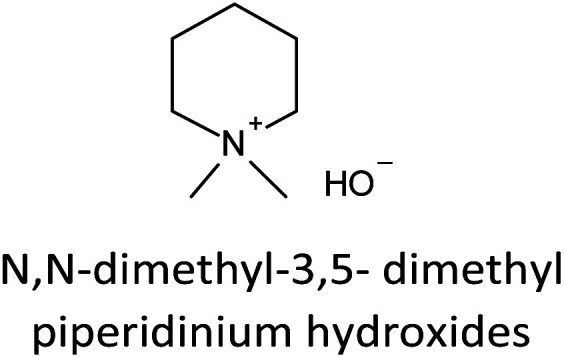	
AFX^137^	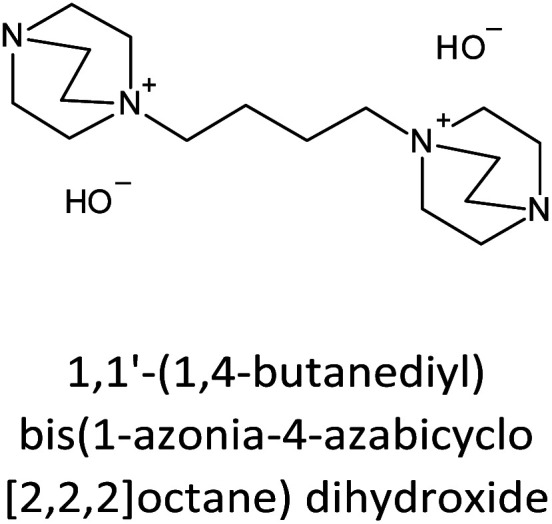	
LEV^122^	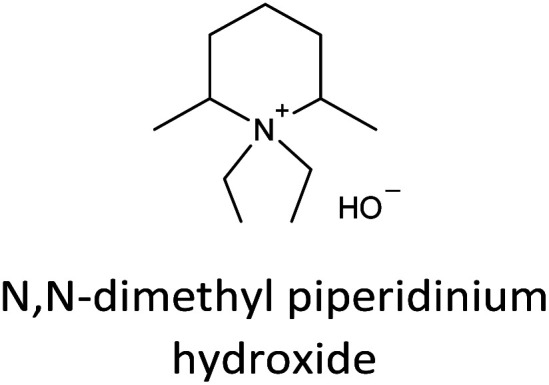	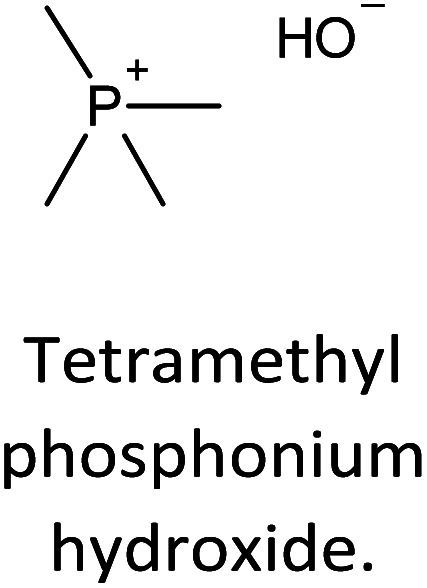

Besides, ^31^P MAS NMR spectroscopy is one of the most used techniques to study the phosphorus species located inside zeolite channels and their evolution during heating treatments. On the right side of Fig. 9, the resonance centered at 40 ppm corresponding to tetraethylphosphonium molecules located inside zeolite AFX cavities disappeared upon sample calcination. At the same time, a complex mixture of resonances between −10 and −40 ppm appeared, corresponding to different P-species interacting with the zeolitic framework.

To achieve a further understanding of these complex spectra, Zhao *et al.*^140^ calculated the theoretical ^31^P NMR chemical shifts of extra-framework phosphorus species for the P-CHA system. The authors studied several proposed models of phosphate species interacting with Brønsted acid sites, finding some possible models that could explain the experimental ^31^P and ^27^Al MAS NMR spectra. These findings improve the assignment of the ^31^P NMR resonances in these types of complex systems where broad signals are frequently observed due to the overlap of resonances corresponding to different species and chemical environments.

#### 5.1.2. Stabilization of zeolites by P incorporation

The acid treatment with a phosphorus precursor and subsequent calcination of the solid lead to the breaking of some Si–O(H)–Al bonds, extraction of Al from the framework and partial amorphization of the structure. The degree of dealumination seems to be related to the content of extra-framework incorporated phosphorus, being essential to finding the optimum P content where the minimum number of acid sites is lost keeping the maximum stability for the framework aluminum.^30,122,141^

Danisi *et al.*^141^ reported the significant influence of the P content in ZSM-5; at a low phosphorus content, P/Al < 1, a partial dealumination and a decrease of the unit cell volume of 0.25% were observed compared to the parent H-ZSM-5. Meanwhile a high P content, P/Al > 1, leads to more dealumination resulting in the relaxation of the framework and transformation from orthorhombic to monoclinic. This effect agrees with previous studies where it was reported that the orthorhombic/monoclinic phase transformation strongly depends on the chemical composition of the framework and temperature, among other factors.^142^

**Fig. 10:**
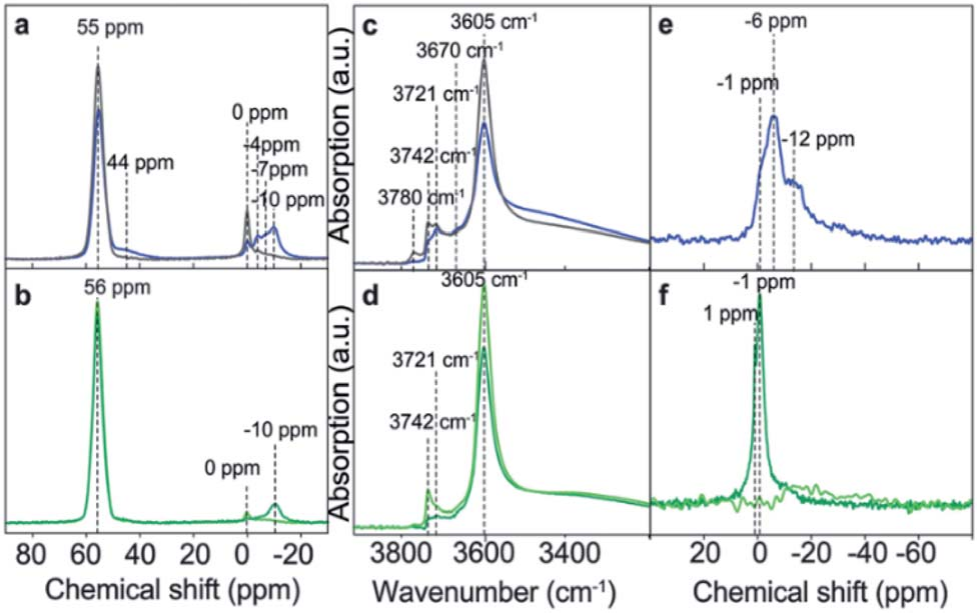
(a and b) ^27^Al MAS NMR spectra and (c and d) FT-IR spectra, highlighting the OH-stretch region, and (e and f) ^31^P MAS NMR spectra of the following samples: H-ZSM-5 impregnated with an aqueous H_3_PO_4_ solution (blue) and after washing with hot water (gray), H-ZSM-5 impregnated with an aqueous (NH_4_)_2_HPO_4_ solution (dark green), and after washing with hot water (light green). Reproduced from ref. 143 with permission from the PCCP Owner Societies.

The presence of phosphorus species modifies the chemical environment of the Al atoms, as can be observed by ^27^Al MAS NMR spectroscopy (Fig. 10). Meanwhile the intensity of the characteristic resonance corresponding to tetrahedral framework Al, centered at 55 ppm, decreases with the P content and at the same time the resonances related to distorted tetrahedral and octahedral coordinated Al, appearing between 0 and −10 ppm, increase.^143^ This P–O–Al interaction is partially reversible, being possible to revert most of the Al to their original tetrahedral coordination by removing the phosphorus species by washing with hot water. However, if a calcination step is performed, the P–O–Al interaction becomes permanent that causes difficulty in the removal of the extra-framework P, which results in the formation of local silicoaluminophosphate interfaces based on the obtained results from ^27^Al and ^31^P MAS NMR and K-edge XANES.^143^

The effect of phosphorus incorporation has been widely studied for the medium pore size MFI zeolite; furthermore, other phosphated 10R structures, such as IMF,^144^ MWW,^121,124^ MEL^145^ and ITH^146^ have shown enhanced stability against dealumination under steaming conditions.

On the other hand, the stabilization effect of the phosphorus in large pore zeolites *BEA and MOR is still not clear and it seems that thermal treatment after phosphorus incorporation leads to the partial dealumination and formation of extra-framework aluminophosphates.^147,148^

**Fig. 11:**
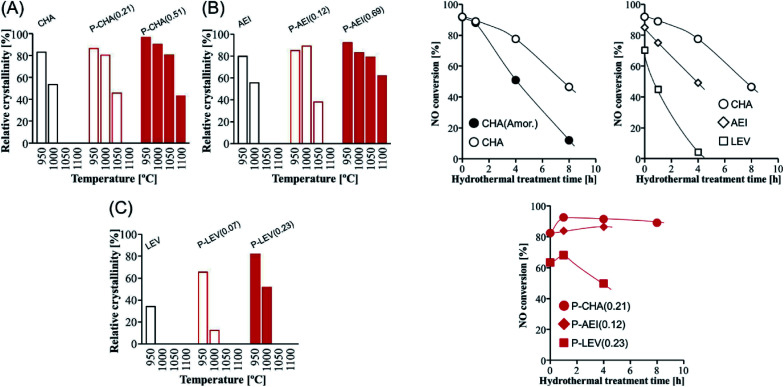
(Left) Relative crystallinity of (A) CHA, (B) AEI, and (C) LEV with and without phosphorus modification after calcination at various temperatures for 1 h. (Right) NO conversion at 200 °C *vs.* the hydrothermal treatment time over Cu-loaded small-pore zeolite catalysts with and without phosphorus modification. Catalyst = 0.8 g (1.5 mL), Cu content = 1.5 wt%, and flow rate = 1.5 mL min^−1^ (200 ppm NO, 200 ppm NH_3_, 10% O_2_, 3% H_2_O, and balanced N_2_). Reprinted from ref. 122, with permission from Elsevier.

The dual-template method developed by Sano *et al.* introduced phosphorus species into small pore zeolites CHA,^136^ AEI,^135^ LEV^122^ and AFX,^137^ controlling the phosphorus content by adjusting the synthesis conditions and opening the study of the influence of the presence of extra-framework phosphorus species in 8R aluminosilicates. In general, it was observed that the presence of phosphorus avoids the structural collapse of these zeolites after thermal treatments at high temperatures. Zeolites CHA and AEI with a P/Al ratio of 0.51 and 0.69, respectively, presented very high stability, with a relative crystallinity higher than 80% after thermal treatment at 1050 °C (Fig. 11).^122,135,136^ Meanwhile zeolites LEV (P/Al = 0.23) and AFX (P/Al = 0.20) showed a relative crystallinity close to 60% after thermal treatment at 1000 °C and 900 °C, respectively.^122,137^ The hydrothermal stability of Cu-containing AEI, CHA and LEV having different P contents was evaluated by a NH_3_-SCR reaction.^122^ NO conversion achieved for CHA, AEI and LEV catalysts in the absence of P decreases with the time of hydrothermal treatment at 900 °C in a stream of 10 vol% H_2_O and 90 vol% air. Meanwhile the presence of P enhances the stability of the zeolites, keeping the NO conversion at high levels, especially in the case of the CHA zeolite, where a NO conversion higher than 80% is observed after 8 hours of hydrothermal treatment, as shown in Fig. 11. The presence of P species not only preserved the zeolite against dealumination but also suppressed the Cu aggregation into CuO_*x*_ clusters after the hydrothermal treatment.^149^

Recently, Simancas *et al.*^150^ reported the direct synthesis of 8- and 10-ring channel aluminosilicate IFW by using a P-containing OSDA. The presence of extra-framework phosphorus species not only stabilizes the Al in framework positions under steaming conditions at 700 °C but also enhances the catalyst performance in the methanol to olefins reaction.

#### 5.1.3. Interaction of P and Al

**Fig. 12:**
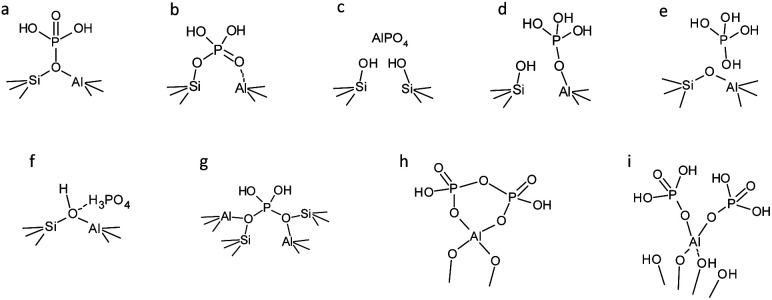
Proposed models of phosphates interacting with the framework (a) Kaeding *et al.*,^173^ (b) Lercher *et al.*,^31^ (c) Caro *et al.*,^174^ (d) Zhuang *et al.*,^175^ (e) Blasco *et al.*,^30^ (f) Abubakar *et al.*,^176^ (g) Xue *et al.*,^177^ (h) van der Bij *et al.*,^143^ and (i) Louwen *et al.*^151^

Although the interaction between extra-framework phosphorus species and framework Al is not fully understood, several models of phosphates interacting with zeolites have been proposed (Fig. 12). Recently, theoretical calculations have become a powerful ally to understand the mechanism of dealumination of zeolites and the stabilization promoted by phosphate incorporation. Louwen *et al.*^151^ studied the energy of the reported models in the literature and proposed another model based on the results after localizing the phosphate groups in a P-containing ZSM-5 zeolite by using synchrotron-based powder XRD, neutron diffraction, pair distribution function (PDF) analysis and quantum-mechanical modeling (QMM). The study concluded that the models proposed by Blasco and Abubakar, and Louwen (Fig. 12e, f and i, respectively) were the most energetically favored.

### 5.2. Incorporation of extra-framework metal cations

#### 5.2.1. Transition metal Cu^2+^ cations

Extra-framework metal transition cations are not only active sites in several reactions, but also can contribute to the framework stability of the zeolite. Upon Cu-SSZ-13 materials commercialization as active and robust catalysts in the NH_3_–SCR process, detailed investigations regarding the Al distribution in the zeolite, Cu cation loadings and nature of active sites have been carried out.^152^

**Fig. 13:**
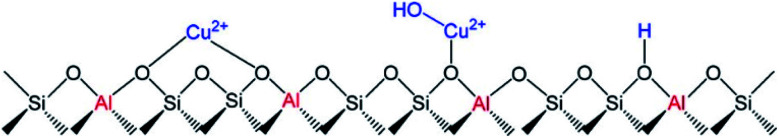
Schema of Cu^2+^–2Z and [Cu(OH)]^+^–Z species and Brønsted acid sites in Cu-SSZ-13. Reprinted with permission from ref. 153. Copyright (2017) American Chemical Society.

Isolated Cu^2+^ cations can be found in two locations in the Cu-SSZ-13 zeolite as shown in Fig. 13. Cu^2+^ balancing 2Al (Cu^2+^–Z) is located in the d6r and [Cu(OH)]^+^ interacting with 1Al ([Cu(OH)]^+^–Z) is located in the CHA cages.^81,153^ By using DFT simulations, Song *et al.*^153^ described the higher hydrothermal stability of Cu^2+^–2Z than [Cu(OH)]^+^–Z in the Cu-SSZ-13 zeolite under SCR reaction conditions, which agrees with the experimental results. [Cu(OH)]^+^–Z species were proposed to be responsible for the Cu agglomeration by hydrolysis and migration through the channels forming small clusters that remain in the pores of the zeolite, confirming the absence of those species on the external surface of the solids by XPS and STEM mapping analysis.

Several zeolite topologies have been tested in NH_3_–SCR reactions but small pore zeolites such as AEI,^154^ AFX,^155^ CHA,^156^ ERI,^155^ KFI,^157^ LTA^158^ and RTH^159^ showed superior hydrothermal stability. The stabilization of the Cu^2+^ cation in the double-6-ring (d6r) framework unit in CHA^156^ and AEI^154^ zeolites has been shown to be an effective way to enhance the stability properties of the zeolites under steaming conditions and mitigate the dealumination process.

Zeolite LTA showed a similar behavior when the catalyst was fully copper-exchanged. Ryu *et al.*^160^ claimed that Cu^2+^ cations are exclusively located in the single-6-ring (s6r) units, eliminating the requirement of the d6r units in the framework of the catalysts useful for NH_3_–SCR reactions with good stability properties.

In the case of the Cu–RTH zeolite, the relatively high framework stability after hydrothermal aging at 750 °C was explained by the limited migration of Cu to other locations, keeping good NH_3_–SCR performance.^159^ Cu cations, which are commonly incorporated by post-synthesis ion exchange treatments, can also be incorporated by direct synthesis. Cu-SSZ-13 zeolites have been prepared by using a copper–amine complex (Cu–tetraethylenepentamine) as the SDA and Cu source,^161,162^ which upon calcination treatment decomposes forming CO_2_, NO_*x*_ and H_2_O that diffuse through the zeolitic channels, whereas Cu^2+^ cations remain inside the cavities of the zeolite.^162^

Cu^2+^ cations protect framework Al against dealumination under steaming conditions; however, the hydrothermal stability of the catalyst decreases at high Cu loadings due to the trend of [Cu(OH)]^+^–Z species to aggregate forming CuO_*x*_ clusters under harsh conditions.^76,161,163^ That behavior highlights the importance of the optimization not only of the Cu content but also the cation location in the extra-framework sites and the interaction with isolated or pair Al sites.

#### 5.2.2. Rare earth and alkaline cations

The incorporation of extra-framework cations has been proven as an efficient approach to enhance the hydrothermal stability of Al-rich zeolites. The stabilization effect on the zeolite structure of extra-framework rare earth cations has been mainly studied in large pore zeolite Y,^28,29^ where the introduction of extra-framework La, Ce, Nd, Sm, Gd, Tb, Dy, Ho, Er, and Tm has shown an enhancement of catalyst thermal stability.^164^ Rare earth cations located in the supercages of zeolite Y, generally, migrate to more stable sodalite cages during calcination, enhancing framework stability. Du *et al.*^165^ observed that a decrease in the cation radii causes a more pronounced stabilization effect. Small cations La, Pr and Nd were mainly found in the site I′ in the SOD cage, whereas Ce was also located in the supercages forming CeO_2_ that inhibits the migration of the cation. Louwen *et al.*^166^ found that the activation energy for zeolite Y dealumination was higher in the presence of La^3+^ cations. The study was done with a realistic model of the Y zeolite, where the Al distribution matches with previously reported studies by NMR spectroscopy and differs from the thermodynamically most stable distribution, which highlights the importance of knowing the local environment of the active sites.

The stabilization effect of the introduction of rare earth cations into small pore zeolites has been less explored probably because of the limited diffusion of the relatively large hydrated rare earth ions through the 8R windows. The incorporation of Ce, La, Sm, Y and Yb into the Cu-SSZ-13 zeolites has been reported to enhance the framework stability after hydrothermal aging at 800 °C.^161,163,167,168^Table 2 shows some recent examples of extra-framework cations used to improve zeolite stability under hydrothermal conditions. Wang *et al.*^163^ reported the incorporation of Ce^3+^ cations to stabilize the Al in framework positions in the zeolite Cu-SSZ-13 after hydrothermal treatment at 800 °C for 20 h; moreover, the H_2_–TPR results suggested that the Cu^2+^ cations in the aged Ce-containing samples are mainly located in the 6R, suggesting that the presence of Ce prevents the migration of Cu^2+^ during the steaming treatment.

**Table 2:** Recent examples of the incorporation of extra-framework rare earth and alkali and alkaline earth metal cations to improve zeolite stability under hydrothermal treatment

Zeolite	Cation	Treatment	Comments	Ref.
USY	La, Ce, Pr, Nd	100% steam at 800 °C for 2–18 h	Crystallinity retention after steaming follows the order CeY < LaY < PrY < NdY	165
Cu-SSZ-13 (CHA)	Ce	10 vol% water vapor in Ar at 720 °C for 10 h (aging) and 800 °C for 20 h (deep aging)	Ce_0.93_Cu_5.65_-SSZ-13 prepared for 2 hours of exchange showed the best hydrothermal stability and catalytic performance	163
Cu-SSZ-13 (CHA)	Ce	10 vol% water vapor in air at 700, 750, 800 and 850 °C for 5 h	A small amount of Ce (0.2–0.4 wt%) improved the hydrothermal stability of Cu-SSZ-13 (Si/Al = 6.5 and Cu content of 3.4–4.1 wt%) at the same time keeping high activity in the NH_3_–SCR reaction	167
Cu-SSZ-13 (CHA)	Ce, La, Sm, Y, Yb	10 vol% water vapor in air at 800 °C for 16 h	Cu_2.8_Y_1.3_-SSZ-13 is the optimum catalyst composition for NH_3_–SCR activity and hydrothermal stability	168
Cu-SSZ-13 (CHA)	Ce, Sm	10 vol% water vapor in air at 800 °C for 8 h	Enhancement of stability by introducing a small amount of Ce or Sm (0.3–0.7 wt%) into Cu-SSZ-13 with a Cu/Al ratio from 0.10 to 0.16	161
Cu-SSZ-13 (CHA)	Li, Na, K, Cs, Mg, Ca	10 vol% water vapor in air at 750 °C for 16 h	Li, Na, K, Cs, Mg, and Ca inhibit Cu-SSZ-13 dealumination	169
Cu-SSZ-13 (CHA)	Na	10 vol% water vapor in air at 750 °C or 800 °C for 5 h	Cu_2.7_Na_1.7_-SSZ-13 is the optimum catalyst composition for NH3–SCR activity and hydrothermal stability	170
Cu-SSZ-13 (CHA)	Na	10 vol% water vapor in air at 750 °C for 16 h	Cu_3.9_Na_0.8_-SSZ-13 is the optimum catalyst composition for NH_3_–SCR activity and hydrothermal stability	171

Zhao *et al.*^168^ studied the effect of incorporation of several rare earth cations (Ce, La, Sm, Y, and Yb), finding that Y^3+^ cations led an enhancement of the hydrothermal stability and catalytic performance of Cu–CHA in the NH_3_–SCR reaction. However, an excess of Y^3+^ causes difficulties in the incorporation and diffusion of Cu^2+^ cations and reduces the activity of the catalyst at low temperatures in the SCR reaction. The preferential location of Y^3+^ in the 8R was confirmed by two-dimensional ^23^Na MQ MAS NMR measurements of Na-CHA and Na,Y-CHA, which showed that the signal corresponding to Na located in the 8R decreases for the sample Na,Y-CHA.

The optimum content of rare earth cations is an important parameter. Usui *et al.*^167^ studied the effect of different Ce loadings in the Cu-SSZ-13 zeolite introduced by standard wet ion exchange using a cerium acetate aqueous solution and by solid-state ion exchange by mixing cerium acetate monohydrated with Cu-SSZ-13 and subsequently calcinating. The location of Cu and Ce in the structure was studied by several characterization techniques, concluding that the Cu^2+^ cations are found in the chabazite cage next to the six-membered rings, while the Ce^3+^ cations are located in the center of the CHA cage.

The enhancement of the Cu-SSZ-13 hydrothermal stability at 850 °C in the presence of a small Ce content (0.2–0.4 wt%) was explained by two possible effects: (1) the cerium cations fill the framework defects protecting the structure and (2) the superior framework stability of the presence of Ce cations on ion-exchange sites instead of protons.

Wang *et al.*^161^ also studied the effect of low loadings of Ce and Sm (0.3–0.7 wt%) in Cu-SSZ-13; in this case, the zeolite was prepared by direct synthesis using the complex Cu-tetraethylenepentamine as the structure-directing agent (SDA) and Cu source simultaneously, while Ce and Sm were incorporated by standard ion exchange. The samples modified with Ce and Sm presented a higher fraction of Cu^2+^–2Z species that are more stable than [Cu(OH)]^+^–Z (see Section 5.2.1) under steaming conditions, suggesting that the presence of a small amount of Ce and Sm plays an important role in the stability of Cu^2+^ cations while the contribution to the Al stabilization is limited.

Despite the increasing number of studies, the explanation for the stability enhancement is still not clear. While some authors concluded that the presence of RE mainly stabilizes the zeolite framework^167^ and limits the dealumination process,^168^ some others claim that the presence of RE inhibits the agglomeration of Cu^2+^ into CuO_*x*_ clusters.^161^ According to a previous study, the formation of CuO_*x*_ clusters is the main contribution of the structural collapse of the catalysts during hydrothermal aging.^153^

Alkali and alkaline earth metals have also been used to improve the hydrothermal stability of zeolites. Gao *et al.*^169^ observed that the presence of co-cations, Li^+^, Na^+^, K^+^, Cs^+^, Mg^2+^ and Ca^2+^, prevents the hydrolysis of Si–(OH)–Al bonds of the zeolite Cu-SSZ-13, reducing the dealumination process of the zeolite after treatment in 10% water vapor in an air flow at 750 °C for 16 h. Zhao *et al.*^170^ also reported the enhancement of framework stability against the dealumination of Na-containing Cu-SSZ-13 under steaming conditions. However, a further increase of the cation content resulted in the collapse of the structure due to the agglomeration of the active Cu species into CuO_*x*_ clusters.^170–172^

## 6. Summary and outlook

Zeolites have been and are expected to be used as catalysts and adsorbents in various processes. In this review, we introduced the recent research topics focusing on the framework stability improvement of zeolites. The precise control of zeolites at the atomic scale such as structural defects and Al location has a significant effect on stability, while the incorporation of extra-framework species such as phosphorus oxides and metal cations is also crucial.

The Al distribution in the framework and the location of the extra-framework phosphorus and Cu cation species showed different stabilization energy, highlighting the importance of the detailed knowledge of the framework to understand and develop new and optimal treatments to enhance the stability. Theoretical studies are potent allies to study those effects and help to explain the experimental results.

The optimization of the improvement of hydrothermal stability strategies described in the review together with the extension of the application to a majority of zeolitic frameworks should open up new applications of zeolites.

## Conflicts of interest

There are no conflicts to declare.
